# Supplementary material for: Phantom of the forest or successful citizen? Analysing how Northern Goshawks (Accipiter gentilis) cope with the urban environment
Source: R Soc Open Sci. 2020 Dec 23;7(12):201356. doi: 10.1098/rsos.201356 (PMC7813232; doi:10.1098/rsos.201356)
Supplement: Electronic supplementary material from Phantom of the forest or successful citizen? Analysing how Northern goshawks (Accipiter gentilis) cope with the urban environment [file rsos201356supp1.pdf]

Supplementary material

From: Phantom of the forest or successful citizen? Analysing how Northern goshawks (*Accipiter gentilis*) cope with the urban environment

Diet composition

Genetic identification of prey remains

To be able to extract the DNA from the found prey items, we first investigated the samples. From the bones we tried to use tissue that was left over. If this was not possible we crushed the dried bones to flour and used this. The found pellets were controlled systematically trying to distinguish different species present in the pellet. Every potential samples from different species were used for further DNA extraction. DNA was extracted using the Dneasy Blood & Tissue Kit (Qiagen, Hilden, Germany) according to the manufacturer's description. The following PCR was conducted using the FastStartTaq DNA Polymerase dNTPack (Roche Diagnostics, Mannheim, Germany) with two primer pairs; on the one hand, CB1-L (5´-CCATCCAACATCTCAGCATGATGAAA-3´) with CB2-H (5´-CCCTCAGAATGATATTTGTCCTCA-3´) [1] and on the other hand, GluDG-L (5´-TGACTTGAARAACCAYCGTTG-3´) [2] with CB2-H.

PCR mixtures contained 0.2 U FastStartTaq DNA Polymerase, 1x FastStart Reaction Buffer, 2.5 mM $MgCl_2$, 200 µM dNTPs, 24 pmol of each primer, and 50 – 100 ng of DNA in a final volume of 25 µl. Reaction mixtures were subjected to the following PCR cycling protocol: 95°C for 10 min, 35 cycles of 94°C for 45 sec, 56°C for 45 sec, and 72°C for 2 min, followed by 72°C for 10 min.

The PCR products were purified (ExoSAP; Thermo Fisher Scientific, Waltham, USA), directly sequenced using the BigDye Terminatorv1.1 Cycle Sequencing kit (Thermo Fisher Scientific, Waltham, USA) and the PCR primers, and analysed on a 3130xl Genetic Analyzer

(Thermo Fisher Scientific, Waltham, USA). Raw sequences were manually inspected and refined using MEGA6 [3] and subsequently aligned to each other and searched against the NCBI nucleotide database [4]. A list of all identified prey species can be found in Table S1.

Statistical analysis

Description of the statistical models

We initially fitted all models accounting for spatial autocorrelation (considering the geographic coordinates as random effects whose realisations depend on the Matérn correlation structure). However, since model fits considering the location as a simple random predictor led to similar results, we only present such model fits for the sake of simplicity.

We also initially computed the percentage of impervious ground (soil sealing) to characterize the level of urbanisation [5]. The percentage was computed within the circular area with a radius of 2.5 km around each nest--corresponding roughly to the territory size of an individual of unknown origin [6-10]. We decided to use such a fixed radius not to bias the assessment of imperviousness, although goshawks tend to have smaller territories in urban than in rural habitats [6-10]. The average imperviousness was 55% (range: 11.0%-81.7%, n = 96) in the urban habitat and 5.1% (range: 0.2%-26.7%, n = 100) in the rural one. Because the distribution of imperviousness measurements only marginally overlapped between urban and rural habitats (Fig. S1), we decided to use the habitat type (rural vs urban) as a binary predictor in all analyses instead of imperviousness for the sake of simplicity. We checked that this simplification had no qualitative effect on our conclusions by running all analyses using either predictors (data not shown).

Behavioural responses

Because attack reactions were extremely rare (see Results), we pooled cases of “alarm calling”, “feint attack” and “physical attack” together, resulting in a binary response variable describing if the mother reacted to our presence (“yes”) or not (“no”). We studied the

influence of independent variables on this binary response using a logistic (binary) Generalised Linear Mixed-effects Model (GLMM). We considered as fixed-effect predictors: the habitat in interaction with categorical age (young or old), the year, as well as other recorded variables which we thought could influence the behaviour in the Northern goshawk – the total amount of rainfall during the sampling day, laying day and number of nestlings. All independent variables but the habitat and the age categorical were considered as quantitative predictors. In terms of random predictors, we included the territory (116 levels) and the location (7 levels).

Diet composition

To investigate difference in the diet between urban and rural Northern goshawks, we fitted three different models: (1) a logistic (binomial) GLMM predicting the proportion of hunted prey that were pigeons; (2) a Linear Mixed-effects Model (LMM) predicting the Simpson index – a measure of diversity [11] – and (3) a zero-truncated negative binomial GLMM predicting species richness. In all three models, the predictors considered were the habitat type (fixed-effect) and the location (6 levels; random-effect). For the first model, we also included an observation-level random effect to account for the presence of over-dispersion [12].

Breeding performance

We predicted the *laying date* using a LMM considering the habitat type and the average temperature during the two months preceding breeding (February and March) as fixed-effect predictors, as well as territory (132 levels) and location (7 levels) as random predictors. Although the laying day is a discrete variable, the Gaussian approximation of the response did not trigger any substantial violation from model assumptions. Due to a strong collinearity between the year and temperature variables, we did not consider year in the model. We controlled for temperature because cold weather is expected to delay the laying of eggs [13-15].

We predicted the *brood size* using a logistic (binary) GLMM with the response variable distinguishing “small brood” (1–2 nestlings) from “large brood” (3–5 nestlings). Despite implying a loss of information, attempts to model the number of nestling directly (using a zero-truncated Poisson distribution) showed strong under-dispersion. Furthermore, the distribution of the number of nestlings appeared bounded on both sides and no simple solution exists to handle such kind of distributions while working on the original counts. The median number of nestlings were 3 in the urban and rural habitat. To obtain comparable sizes per category the categories were thus split into the two following groups: 1-2 nestlings and 3-5 nestlings. In the logistic GLMM, we considered the habitat type, the laying date of the first egg and the average temperature during the two months preceding breeding as fixed-effect predictors, and the territory (132 levels) and the locations (7 levels) as random predictors. Year was excluded from the model for the same reasons as in the previous model.

Health status

To investigate the infection rate and the chance to develop clinical signs of the disease, we used logistic (binary) GLMMs with the presence/absence of *T. gallinae* as well as the presence/ absence of clinical signs as response variables. As predictors, we included in both models the habitat type, sex of the nestlings, age of the nestlings, number of nestlings, year, temperature and laying day. All fixed-effect predictors were continuous but the sex and habitat. Again, we considered the territory (133 levels) and the location (7 levels) as random predictors.

Causes of mortality

To further investigate the health threat for the Northern goshawk populations, we compared the frequencies of the causes of death having the potentially the largest impact on urban goshawk populations – trichomonosis-related causes of death and collisions with windows. To do this, we fitted two logistic (binary) Generalised Linear Models (GLM). We considered

as fixed-effect predictors: the habitat and sex. In the trichomonosis model, we included the age of the dead bird as well, because trichomonosis is known to mainly infect nestlings [16].

Reference list

1 Kocher, T. D., Thomas, W. K., Meyer, A., Edwards, S. V., Pääbo, S., Villablanca, F. X., Wilson, A. C. 1989 Dynamics of mitochondrial DNA evolution in animals: amplification and sequencing with conserved primers. *Proceedings of the National Academy of Sciences*. **86**, 6196-6200.

2 Palumbi, S., Martin, A., Romano, S., McMillan, W., Stice, L., Grabowski, G. 1991 The simple fool's guide to PCR, ver. 2.0. *Honolulu: University of Hawaii*. 46.

3 Tamura, K., Stecher, G., Peterson, D., Filipski, A., Kumar, S. 2013 MEGA6: Molecular Evolutionary Genetics Analysis version 6.0. *Mol Biol Evol*. **30**, 2725-2729. (10.1093/molbev/mst197)

4 Altschul, S. F., Gish, W., Miller, W., Myers, E. W., Lipman, D. J. 1990 Basic local alignment search tool. *Journal of Molecular Biology*. **215**, 403-410. (https://doi.org/10.1016/S0022-2836(05)80360-2)

5 Langanke, T., Büttner, G., Dufourmont, H., Iasillo, D., Probeck, M., Rosengren, M., Sousa, A., Strobl, P., Weichselbaum, J. 2015 GIO land (GMES/Copernicus initial operations land) High Resolution Layers (HRLs)–summary of product specifications. *European Environment Agency*.

6 Kenward, R. E. 1982 Goshawk hunting behaviour, and range size as a function of food and habitat availability. *J Animal Ecol*. **51**, 69-80. (10.2307/4311)

7 Kenward, R. E. 1976 The effect of predation by goshawks, Accipiter gentilis, on Wood pigeon, Columba palumbus, populations: University of Oxford.

8 Ziesemer, F. 1983 *Untersuchungen zum Einfluss des Habichts (Accipiter gentilis) auf Populationen seiner Beutetiere: erweiterte Fassung eines Gutachtens für die Oberste Jagdbehörde des Landes Schleswig-Holstein*. Kronshagen: Hartmann.

9 Würfels, M. 1994 Entwicklung einer städtischen Population des Habichts (Accipiter gentilis) und die Rolle der Elster (Pica pica) im Nahrungsspektrum des Habichts—Ergebnisse vierjähriger Beobachtungen im Stadtgebiet von Köln. *Charadrius*. **30**, 82-93.

10 Rutz, C. 2001 Raum-zeitliche Habitatnutzung des Habichts-Accipiter gentilis-in einem urbanen Lebensraum: Diploma thesis, University of Hamburg, Hamburg, Germany.

11 Whittaker, R. H. 1972 Evolution and measurement of species diversity. *Taxon*. **21**, 213-251. (10.2307/1218190)

12 Harrison, X. A. 2015 A comparison of observation-level random effect and Beta-Binomial models for modelling overdispersion in Binomial data in ecology & evolution. *PeerJ*. **3**, e1114. (10.7717/peerj.1114)

13 Looft, V., Biesterfeld, G. 1981 Habicht–Accipiter gentilis. *Vogelwelt Schleswig-Holsteins*. **2**, 101-115.

14 Bijlsma, R. 1993 *Ecologische atlas van de roofvogels van Nederland*. Haarlem: Schuyt & Co. [In Dutch, English summary.].

15 Kenward, R. 2006 *The goshawk*. London: T&AD Poyser/A&C Black.

16 Urban, E. H., Mannan, R. W. 2014 The potential role of oral pH in the persistence of Trichomonas gallinae in Cooper's Hawks (Accipiter cooperii). *J Wildl Dis*. **50**, 50-55. (10.7589/2012-12-322)
