# Supplementary figures and images for: Phantom of the forest or successful citizen? Analysing how Northern Goshawks (Accipiter gentilis) cope with the urban environment
Source: R Soc Open Sci. 2020 Dec 23;7(12):201356. doi: 10.1098/rsos.201356 (PMC7813232; doi:10.1098/rsos.201356)

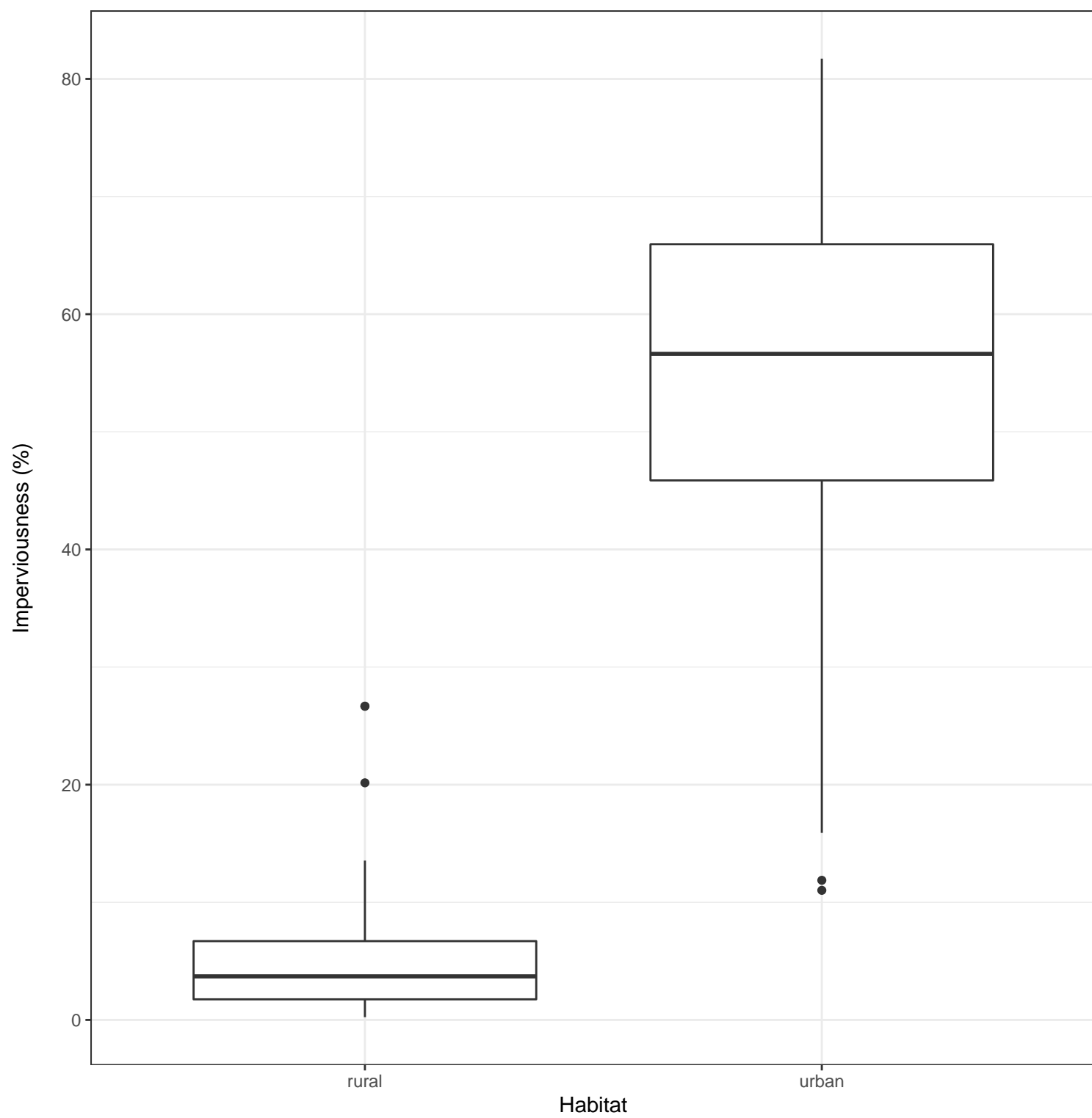

Supplement: Figure S1 Imperviousness Index from Phantom of the forest or successful citizen? Analysing how Northern goshawks (Accipiter gentilis) cope with the urban environment [file rsos201356supp2.pdf]

Number of species

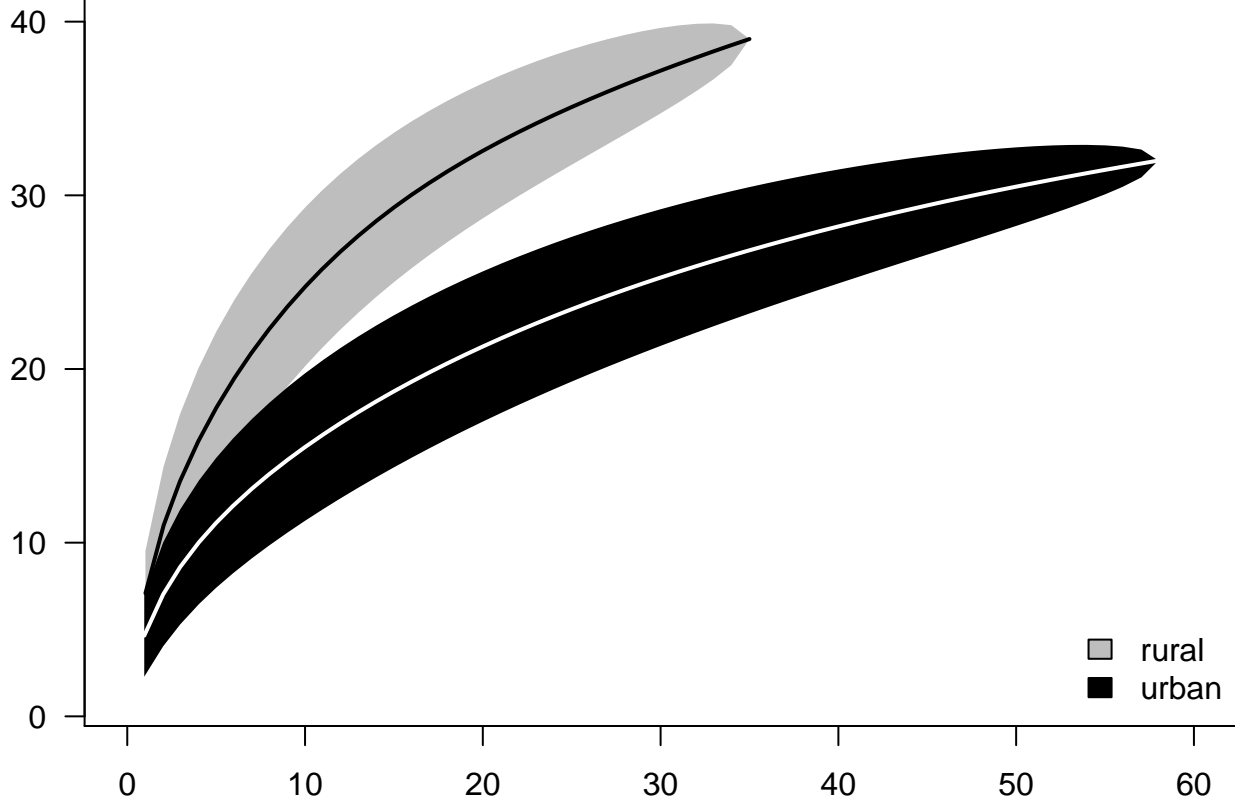

Territory

■ rural  
■ urban

Supplement: Figure S2 Rarefaction curves from Phantom of the forest or successful citizen? Analysing how Northern goshawks (Accipiter gentilis) cope with the urban environment [file rsos201356supp3.pdf]
