# Supplementary material for: Phantom of the forest or successful citizen? Analysing how Northern Goshawks (Accipiter gentilis) cope with the urban environment
Source: R Soc Open Sci. 2020 Dec 23;7(12):201356. doi: 10.1098/rsos.201356 (PMC7813232; doi:10.1098/rsos.201356)
Supplement: Table S1 Single prey species from Phantom of the forest or successful citizen? Analysing how Northern goshawks (Accipiter gentilis) cope with the urban environment [file rsos201356supp4.pdf]

Table S1: Amount of all prey species in urban and rural habitats.

Species name		Percentage by number of occurrence [%]	
		Urban	Rural
BIRDS			
Feral pigeon	*Columba livia domestica*	44.69	20.18
Woodpigeon	*Columba palumbus*	19.05	13.45
Eurasian magpie	*Pica pica*	8.61	1.46
Carrion crow	*Corvus corone*	6.04	1.46
Common blackbird	*Turdus merula*	4.40	8.77
Common starling	*Sturnus vulgaris*	3.48	6.14
Eurasian jay	*Garrulus glandarius*	2.38	9.36
European turtle dove	*Streptopelia turtur*	1.28	0
Long-eared owl	*Asio otus*	0.92	0
Hawfinch	*Coccothraustes coccothraustes*	0.73	0
Eurasian woodcock	*Scolopax rusticola*	0.73	0.58
Rose-ringed parakeet	*Psittacula krameri*	0.55	0
Song thrush	*Turdus philomelos*	0.55	2.05)
Mallard	*Anas platyrhynchos*	0.55	1.75
Eurasian blue tit	*Cyanistes caeruleus*	0.37	0.29
Great spotted woodpecker	*Dendrocpos major*	0.37	8.48
Eurasian jackdaw	*Corvus monedula*	0.37	5.85
European green woodpecker	*Picus viridis*	0.37	0
Mistle thrush	*Turdus viscivorus*	0.37	0.58
Eurasian collared dove	*Streptopelia decaocto*	0.37	0.88
Common chaffinch	*Fringilla coelebs*	0.18	2.34
Eurasian siskin	*Spinus spinus*	0.18	0
House sparrow	*Passer domesticus*	0.18	0
Black-headed gull	*Chroicocephalus ridibundus*	0.18	0.29
European robin	*Erithacus rubecula*	0.18	0
Sparrowhawk	*Accipiter nisus*	0.18	1.17
Fieldfare	*Turdus pilaris*	0.18	1.46
Tawny owl	*Strix aluco*	0.18	1.46
Budgerigar	*Melopsittacus undulatus*	0.18	0
Goldcrest	*Regulus regulus*	0.18	0
Eurasian coot	*Fulica atra*	0	1.46
Northern lapwing	*Vanellus vanellus*	0	1.46
Stock dove	*Columba oenas*	0	1.17
Little owl	*Athene noctua*	0	0.88
Eurasian skylark	*Alauda arvensis*	0	0.58
Black woodpecker	*Dryocopus martius*	0	0.58
Chicken	*Gallus gallus domesticus*	0	0.29
Great tit	*Parus major*	0	0.29

Common raven	*Corvus corax*	0	0.29
Eurasian teal	*Anas crecca*	0	0.29
Redwing	*Turdus iliacus*	0	0.29
European herring gull	*Larus argentatus*	0	0.29
MAMMALS			
European rabbit	*Oryctolagus cuniculus*	1.10	0.29
Norway rat	*Rattus norvegicus*	0.92	1.17
Bank vole	*Myodes glareolus*	0	1.17
Common vole	*Microtus arvalis*	0	0.58
Eurasian harvest mouse	*Micromys minutus*	0	0.29
Eurasian red squirrel	*Sciurus vulgaris*	0	0.29
Yellow-necked field mouse	*Apodemus flavicollis*	0	0.29
